# Bovine oocyte developmental competence and gene expression following co-culturing with ampullary cells: An experimental study

**DOI:** 10.18502/ijrm.v19i4.9063

**Published:** 2021-04-22

**Authors:** Mehdi Azari, Mojtaba Kafi, Anise Asaadi, Zohreh Pakniat, Beheshteh Abouhamzeh

**Affiliations:** ^1^Department of Anatomical Sciences, School of Medicine, AJA University of Medical Sciences, Tehran, Iran.; ^2^Department of Animal Reproduction, School of Veterinary Medicine, Shiraz University, Shiraz, Iran.

**Keywords:** Ampulla, Bovine, Fertilization, Gene expression, IVM.

## Abstract

**Background:**

There is no sufficient information on the impact of bovine ampullary oviductal epithelial cells (BAOECs) on in vitro oocyte maturation competence and gene expression.

**Objective:**

This study aimed to examine the oocyte developmental competence following co-culturing with a monolayer of fresh and frozen-thawed ampullary cells.

**Materials and Methods:**

Bovine cumulus-oocyte complexes (COCs) were distributed into three groups: control group; where in COCs were cultured in cell-free media for 24 hr and FML and FTML groups in which the COCs were cultured in maturation media for 18 hr and then transferred into a media containing fresh and frozen-thawed BAOECs monolayer, respectively (BAOECs were extracted from the oviducts of slaughtered cattle and were then cultured freshly or frozen-thawed) for a further 6 hr. After 24 hr, the expanded COCs were evaluated for nuclear maturation, fertilization rate, and gene expression (*GDF9, StAR, CASP3*, and *FSHr*).

**Results:**

Nuclear maturation rate in the FTML group was significantly higher than the control group (p = 0.02). The fertilization rate of FTML group was significantly higher than the control and FML groups (p = 0.05 and p = 0.03, respectively). In terms of gene expression, *GDF9* were upregulated in the presence of the BAOECs during the last 6 hr of the in vitro maturation (p < 0.001). Furthermore, the expression of the *StAR* gene in the FTML group was higher than the other groups (p = 0.02).

**Conclusion:**

Ampullary cells co-culturing (especially frozen-thawed cells) for in vitro maturation of bovine oocytes yields encourages the results and demonstrates the beneficial effect of co-culture on gene expression and developmental competence.

## 1. Introduction

Despite considerable improvements in the production of bovine embryos in vitro, the quality of embryos is lower than that of the embryos produced in vivo. Optimization of in vitro maturation (IVM), as the primary and critical step, to mimic in vivo conditions could lead to enhanced in vitro embryo production.

The oviduct plays an active role in the reproductive process through its secretion (1-3). The oviductal fluid contains various compounds such as proteins, carbohydrates, ions, and lipids. The amount and composition of oviductal secretions change depending on the different areas of the oviduct at various stages of the estrous cycle (4). The effects of the oviductal epithelial cells and secretion on gamete function and interaction have been investigated in numerous studies (3). This effect depends on the oviductal region and the stage of the estrous cycle. During exposure of oocyte zona pellucida with oviductal fluid, it is modified by different molecules. Sperm-zona binding requires components on the surface of the zona pellucid (such as oviductins and osteopontin) that are acquired in the oviduct (3). Studies have shown that the fertilization rate of hamster and dog oocyte was improved in the presence of oviductal fluid via increased sperm-zona binding and zona penetration (5). Also in bovine and porcine, oviductal fluid increased zona pellucid resistance and reduced polyspermy via decreasing sperm-zona binding (6). One way to improve in vitro embryo production efficiency is using cell co-culturing (7). Co-culturing with oviductal epithelial cells and mesenchymal stem cells improve embryonic development competence. Increased blastocyst rate with high number of blastomeres, high survival rate of post-thaw embryos, high implantation rate, and lower apoptosis have already been indicated in human and animal embryos (8, 9). Besides, the use of fresh primary epithelial cells as feeder co-culturing is impractical for applications due to methodological complication and lack of reproducibility (10). One alternative way to reduce this problem is using stored frozen oviductal epithelial cells for co-culturing systems (10).

Gene expression pattern during oocyte maturation could be an efficient way to study the oocyte development. Correlation between the oocyte and its surrounding cumulus cells is an essential procedure to develop a competent oocyte. Growth differentiation factor 9 (GDF9) and follicle-stimulating hormone receptor (FSHr) are important biomarkers that have a direct effect on cumulus cells functions and oocyte development (11). Caspase-3 is the key caspase family member playing crucial roles in many cell apoptosis events. Steroidogenic acute regulatory protein (*StAR)* gene in cumulus-oocyte complexes (COCs) has been reported to be involved in the developmental competence of the oocyte via biosynthesis of all the steroids molecules and the embryo in bovine (12).

The aim of this study was to investigate the influence of co-culturing bovine oocytes with a monolayer of fresh and frozen-thawed bovine ampullary oviductal epithelial cells (BAOECs) in the last 6 hr of in vitro maturation on maturation and fertilization rate. Furthermore, the expression changes of the genes involved in oocyte maturation and competence (*GDF9, StAR, CASP3*, and *FSHr*) were investigated (experiment 3).

## 2. Materials and Methods

### Experimental design

**Figure 1:**
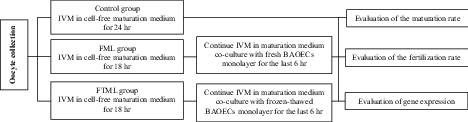
Experimental design. Control: Oocytes cultured in cell-free maturation medium for 24 hr, FML: Oocytes cultured in the cell-free maturation medium for 18 hr and then moved into other wells covered with fresh BAOECs monolayer for the last 6 hr of maturation, FTML: Oocytes cultured in the cell-free maturation medium for 18 hr and then moved into other wells covered with frozen- thawed BAOECs monolayer for the last 6 hr of maturation.

This experimental study was conducted at the IVF laboratory of the School of Veterinary Medicine, Shiraz University, Iran. In group 1 (control, n = 140), the COCs were cultured for 24 hr in cell-free maturation medium; in group 2 (FML, n = 125), the COCs were cultured in the cell-free maturation medium for 18 hr and then moved into other wells covered with fresh BAOECs monolayer for the last 6 hr of maturation; and in group 3 (FTML, n = 146), the COCs were cultured in the cell-free maturation medium for 18 hr and then moved into other wells covered with frozen-thawed BAOECs monolayer for other 6 hr. In experiment 1, in vitro bovine oocyte maturation was performed in cell-free maturation medium for 18 hr and then moved into other wells covered with BAOECs monolayer for the last 6 hr of maturation. The rate of nuclear maturation was evaluated at the end of maturation. In experiment 2, after oocyte maturation same as in experiment 1, oocyte and sperm co-cultured for 18 hr; and aceto-orcein staining used to determine the fertilization rate. In experiment 3, oocytes were matured the same as in experiment 1 and were then used for real-time polymerase chain reaction (PCR) to investigate gene expression patterns (Figure 1). All chemicals were purchased from Sigma (St. Louis, MO, USA), unless otherwise indicated.

### BAOECs isolation and freezing

The oviduct epithelial cells were collected from slaughtered cattle at the beginning of the met-estrus phase of the estrus cycle (i.e., ovarian antral follicles that were ruptured within the last 24 hr). Ampullary epithelial cells were isolated by scraping the mucosal epithelial layer with a sterile glass slide and transferred into the washing medium (TCM-199, gentamicin (40 µg/ml) and amphotericin B (1 µl/ml)). To wash the cells, centrifuging was done twice at 400 g for 5 min. To obtain single cells, Trypsin-EDTA enzyme was added to the sample for 3 min at 37°C and then thwarted using washing media supplemented by 10% fetal calf serum (FCS). Recovered fresh epithelial single cells were seeding in the culture medium [TCM-199, 15% FCS and gentamicin (40 µg/ml)]. The remaining fresh cells were frozen using a slow freezing method (13). Briefly, fresh cells with a concentration of 1 × 10${}^{7}$ cell/ml supplemented with 15% FCS and 10% dimethyl sulfoxide (DMSO) were located in a -20°C freezer for approximately 4 hr and then moved to a -80°C freezer for at least 24 hr. The frozen cells were kept in liquid nitrogen until used. In order to check the quality of cells, all samples of the fresh cells were cultured. Only cells with good-quality monolayer were used for subsequent experiments (14).

### BAOECs thawing and culturing

For thawing cells, five days prior to use, frozen cells were warmed in 37°C sterile water, and the culture media was added to the cells. To remove DMSO, the cells were centrifuged at 400 g for 5 min and re-suspended in warm culture media and cultured into four-well culture dishes (Nunc${}^{\mathrm{TM}}$, Denmark) with a concentration of 1 × 10${}^{6}$cell/ml. During the cell culture, the media was refreshed every 48 hr. One day before co-culturing the oocytes with BAOECs, the culture media was substituted by maturation media (14).

### Oocyte collection

Cattle ovaries were transferred from the local slaughterhouse to the laboratory in saline solution at 35°C, within 3 hr. COCs were aspirated from antral follicles (2-8-mm follicles) with an 18-G needle. COCs with homogenous cytoplasm and intact compact layers of cumulus cells were selected for IVM.

#### Experiment 1: IVM of the bovine oocyte

HEPES-buffered TCM-199 supplemented with 10% FCS and 50 µg/ml gentamicin was used to wash the selected COCs. Bovine oocytes (n = 411) were randomly allocated into three groups. Groups of 30-40 COCs were cultured in four-well culture dishes contained 500 μL equilibrated maturation medium at 38.5°C in 5% CO${}_{2}$ at 90% humidified atmosphere. The maturation medium included of TCM-199 supplemented with 0.1 IU/mL recombinant human FSH (Follitrope, LG Life Sciences, South Korea), 5 IU/mL highly purified hCG (Karma, Pharmatech GmbH, Germany), 50 μg/mL gentamicin, and 10% FCS. This experiment was carried out in five independent replicates (15).

#### Assessment of the IVM

After IVM, the COCs were stripped by frequent pipetting, mounted on glass slides, and fixed in acetic alcohol (1:3) solution for at least 24 hr. On the day of evaluation, 1% aceto-orcein dye (1% orcein in 45% glacial acetic acid) was used to stain the COCc which were mounted on microscopic slides and examined in terms of nuclear morphology with a light microscope at × 100 and × 400 magnifications. Based on their nuclear status, the oocytes were categorized as mature, immature, metaphase І, and abnormal (any spindle and chromosomal abnormality) (15).

#### Experiment 2: In vitro fertilization 

The procedures for IVM were performed as in the first experiment. In group 1 (control, n = 157), group 2 (FML, n = 172), and group 3 (FTML, n = 143), the COCs were matured in vitro as described in Experiment 1. Following the IVM, the COCs were transferred to the fertilization medium (modified Tyrode's medium). In this study, previously tested frozen semen was used for fertilization (15). Motile spermatozoa (obtained through the swim -up method at a final concentration of 10${}^{6}$ spermatozoa ml-1) and the oocytes were co-incubated for 18 hr at 38.5°C in 5% CO${}_{2}$ by applying the highest relative humidity. This experiment was performed in five independent replicates.

#### Evaluation of the fertilization rate

After insemination time (18-20 hr), Presumptive zygotes were denuded and mounted on glass slides under coverslips and fixed for at least 24 hr in acetic alcohol (1:3) solution. For evaluation, 1% aceto-orcein dye was used to stain the presumptive zygotes which were mounted on the slides and tested in terms of fertilization under a light microscope at × 100 and × 400 magnifications. According to their status, presumptive zygotes were categorized as fertilized (presence of two pronuclei), unfertilized ova (without pronuclear conformation and no existence of sperm in the ooplasm and nonappearance of the second polar body), polyspermy (creation of more than two pronucleus), and degenerate (oocytes with other nuclear organization) (16).

#### Experiment 3: Sample collection, RNA extraction, and cDNA synthesis

The procedures for IVM were performed as in the first experiment. “After maturation, 50 COCs in three replicates were gathered from each group and transferred to RNA protectant (Qiagen, Europe) and stored at -70°C until RNA extraction. Total RNA was isolated using RNeasy Micro Kit (cat. no.74004, Qiagen, Hilden, Germany). Total concentration of the extracted RNA was estimated by NanoDrop 2000 spectrophotometer (absorbance at 260 nm wavelength). Sample purity was detected based on the A260/A280 nm ratio, with ratio expected to fall between 1.8 and 2.0. Reverse transcription reactions was performed using a Revert Aid${}^{\mathrm{TM}}$H minus First Strand cDNA Synthesis Kit (K1632, Fermentas, Germany) and random hexamer as a primer, based on the manufacturer's instructions” (11).

#### Real-time polymerase chain reaction

Quantitative RT-PCR was applied to evaluate gene expression according to the previous study. First primers were designed on the primer design software (Perl Primer). Table I lists the expected fragment size and Gen Bank accession numbers. The PCR mix in each well contained of 5 μL of Power SYBRⓇGreen PCR Master Mix (Applied Biosystems, UK), 11 μL dH2O, 1 μL of each forward and reverse primer (5 pmol/μL) for each gene, and 2 μL of cDNA (12.5 ng/μL) in a final volume of 20 μL. PCR reactions were carried out following program: denaturing cycle of 10 min at 95°C, followed by 40 cycles of PCR (95°C for 15 sec, annealing at 60°C for 15 sec, and extension at 95°C for 15 sec). The last step was done with a ramp rate of 2% to generate a melting curve of the product. Each reaction was performed in duplicate, and the mean value of each duplicate was used for more calculations. GAPDH was used as calibrator genes for normalization of target genes. The relative expression of each gene was computed using the ΔΔCT method with efficiency correction. The expression level of each gene in different groups was calculated in relation to the calibrator group (control group) (11).

PCR reactions were carried out following program: Stage 1: 95°C for 10 min. Stage 2: 95°C for 15 s and 60°C for 1 min for in total, 40 cycles. Stage 3: 95°C for 15 s, 60°C for 15 s and 95°C for 15 s. The last heating step in Stage 3 was performed with a ramp rate of 2% to enable the generation of a dissociation curve of the products. Each reaction was run in duplicate, and the mean value of each duplicate was used for the further calculations. The expression data were normalized using the geometric mean of the internal reference genes (GAPDH and YWHAZ). Then, 2-ΔCT (geometric mean of housekeeping genes) values were calculated for each gene.

**Table 1:** Primer sequence and product length


**Gene**	**Primer sequences (5' to 3')**	**Annealing temp. (°C)**	**PCR products (bp)**
	For: TGAAGATATGATAGCCACTAAG	
<brow>-2</erow> *GDF9*	Rev: CTCCTCCTTACACAACAC	<brow>-2</erow> 58	<brow>-2</erow> 170
	For: AACTGGACTGTGGTATTG	
<brow>-2</erow> *CASP3*	Rev: AGGTGCTGTAGAATATGC	<brow>-2</erow> 54	<brow>-2</erow> 104
	For: AATGATGTCTTGGAAGTGATAG	
<brow>-2</erow> *FSHR*	Rev: CGATGTATAGCAGGTTGTTG	<brow>-2</erow> 58	<brow>-2</erow> 98
	For: TGCCGAAGACCATCATCAAC	
<brow>-2</erow> *StAR*	Rev: GAGCCCTCAAACCCATTCAG	<brow>-2</erow> 62	<brow>-2</erow> 157
	For: ATCTCGCTCCTGGAAGATG	
<brow>-2</erow> *GAPDH*	Rev: TCGGAGTGAACGGATTCG	<brow>-2</erow> 60	<brow>-2</erow> 227
*GDF9: Growth differentiation factor 9, CASP3: Caspase 3, FSHr: Follicle-stimulating hormone receptor, StAR: Steroidogenic acute regulatory protein, GAPDH: Glyceraldehyde-3-Phosphate Dehydrogenase*			

### Ethical considerations

This study was certified (IR.AJAUMS.REC.1397.039) by the Ethical and Research Committee of the School of Veterinary Medicine, Shiraz University, Iran.

### Statistical analysis

Data on oocyte nuclear maturation, fertility rate, and gene expression were analyzed using a one-way analysis of variance (ANOVA) and post Tukey test. P-value < 0.05 was considered statistically significant. All statistical analyses were performed using a computer-aided statistical software package (IBMⓇSPSS Statistics version 22, Chicago, IL, USA).

## 3. Results 

### Experiment 1

Table II presents the effect of co-culturing conditions on in vitro nuclear oocyte maturation. The mean (± SE) nuclear maturation rate in the FTML group was higher than that in the control group (p = 0.02). No significant difference was observed in nuclear maturation between the co-culture groups.

### Experiment 2 

The mean ± SE percentage of fertilized oocytes in the FTML group was higher than that in the control and FML groups (P = 0.05 and P = 0.03, respectively) (Table III).

### Experiment 3

**Figure 2:**
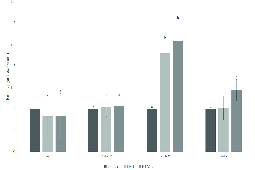
The relative expression levels (Mean ± SD) of *CASP3*, *GDF9, FSHr*, and *StAR*. Different letters in the bars show statistically significant differences (P < 0.05). Data were compared by One-way Analysis of Variance (ANOVA) and post Tukey's test.

The expression of *GDF9* in the treatment groups (FML and FTML groups) was significantly higher than that in the control group (P ≤ 0.001). Furthermore, the expression of *StAR* gene in the FTML group was higher than that in the FML and control groups (p = 0.02). No significant difference was observed among the experimental groups regarding the expression level of *FSHr* and *CASP3* (Figure 2).

**Table 2:** The effects of co-culture fresh and frozen-thawed BAOECs on the nuclear maturation of bovine COCs


**Group**	**N**	**Mature**	**Immature**	**Metaphase I**	**Abnormal**
**Control**	140	94 (67.09 ± 3.5)	28 (20.7 ± 2.6)	9 (5.6 ± 2.7)	9 (6.4 ± 3.1)
**FML**	125	101 (82.2 ± 5.9)	15 (10.3 ± 6.4)	4 (3.1 ± 2.1)	5 (4.2 ± 1.1)
**FTML**	146	129 (89.4 ± 3.7) *	6 (4.01 ± 1.6) **	4 (2.3 ± 1.4)	7 (4.1 ± 2.5)
Data presented as n (Mean ± SE), ${}^{*}$FTML vs control group (P = 0.02), ${}^{**}$FTML cells vs control group (P = 0.01), One-way analysis of Variance (ANOVA) and post Tukey's test, FML: Oocytes cultured in the cell-free maturation medium for 18 hr and then moved into other wells covered with fresh BAOECs monolayer for the last 6 hr of maturation, FTML: Oocytes cultured in the cell-free maturation medium for 18 hr and then moved into other wells covered with frozen-thawed BAOECs monolayer for the last 6 hr of maturation					

**Table 3:** The effects of co-culture on the fertility rate of bovine COCs


**Group**	**N**	**Fertilized**	**Unfertilized**	**Polyspermy**	**Degenerated**
**Control**	157	95 (60.7 ± 4.4)	49 (31.3 ± 2.07)	5 (3.2 ± 1.8)	8 (4.6 ± 3.8)
**FML**	172	101 (59.5 ± 4.04)	48 (28.05 ± 1.2)	10 (5.6 ± 1.5)	13 (6.7 ± 3.8)
**FTML**	143	108 (76.4 ± 4.07)*	20 (13.9 ± 1.3)**	3 (1.7 ± 1.1)	12 (7.8 ± 2.8)
Data presented as n (Mean ± SE), ${}^{*}$FTML vs control group and FML group (P = 0.05 and P = 0.03, respectively), ${}^{**}$FTML vs other groups (P ≤ 0.001), One-way Analysis of Variance (ANOVA) and post Tukey's test, FML: Oocytes cultured in the cell-free maturation medium for 18 hr and then moved into other wells covered with fresh BAOECs monolayer for the last 6 hr of maturation, FTML: Oocytes cultured in the cell-free maturation medium for 18 hr and then moved into other wells covered with frozen-thawed BAOECs monolayer for the last 6 hr of maturation					

## 4. Discussion

According to our search, this is the first study investigating the differences in gene expression of bovine COC following the supplementation with or without monolayer of BAOECs in the last 6 hr of IVM. According to our findings, incubating cattle oocytes with oviductal cells improve oocyte maturation rates and their gene expression profile. After ovulation, bovine COCs complete the maturation process in the microenvironment provided by the oviduct. Kölle and colleagues showed that once the COC enters the oviduct, it attaches to the ampullary portion of the oviductal epithelium by cumulus cells and their matrix (1). In this study, we used fresh and frozen-thawed ampullary cells to mimic in vivo conditions to improve IVM. The use of fresh primary epithelial cells for co-culturing is impractical for the applications due to a methodological complication, lack of repeatability, and bio-sanitary venture (10). Making of a cell bank and the use of frozen cells can make it easy-to-use co-culturing systems. The main finding of our study is that co-culture of BAOECs during the last 6 hr of IVM increases maturation and fertilization rate. In the present study, although no significant difference was observed between the co-culture groups in terms of maturation rate, fertilization rate was higher in the frozen-thawed ML cells group. In the freezing process, the defective cells will be destroyed and only healthy cells will remain. Also in the FTML group, cells with an acceptable quality of monolayer were pooled for subsequent oocyte co-culturing while in the FML group there was variation among the fresh cells obtained from the oviduct of different cattle. However, using frozen-thawed BAOECs, it would be possible to gain the most suitable cell line of BAOECs and use it for the production of bovine IVP. In the previous study, we showed that the co-culture of bovine oocytes with a monolayer of frozen-thawed BAOECs in the last 6 hr of IVM increased both the quantity and quality of the produced embryos (14).

Our finding is consistent with those of previous studies concerning the effect of co-culture on oocyte IVM (17-20). For example, No and co-workers showed that canine oviduct cells from the estrus stage facilitate maturation of canine oocyte and embryonic development (20). In sheep, it was indicated that IVM culture conditions (i.e., co-culture with ampulla oviductal epithelial cells) have a high impact on oocyte development competence and fertilization rate (17, 18). In addition, previous studies revealed that the oviductal cells or fluid of cows in the non-luteal phase of the estrous cycle had a positive effect on in vitro embryo production (21).

Studies indicated that in vitro- matured bovine oocyte has different gene expression patterns in vitro (22, 23). Gene expression patterns of the cumulus and oocytes have been considered as the molecular markers of the oocyte quality (24). Our results demonstrate that the IVM procedure affects the development gene expression profile of cattle oocytes. In our study, the IVM of oocytes co-cultured with ampullary cells improved the oocyte maturation rates. Also, their genes expression profiles are more competent than oocytes that are matured in vitro in a conventional way. *GDF9* is among important oocyte secreted factors (OSFs), which are extremely associated with cumulus cell expansion and future development of COCs. *GDF9* also prevents cumulus cell apoptosis; there by improving oocyte developmental competence (25). In the present research, oocytes in the co-cultured groups expressed a higher level of *GDF9* compared to the control group. *StAR* is undoubtedly a key regulatory protein that transports cholesterol to the inside of the mitochondrial membrane and participates in the rate-limiting step of the biosynthesis of all the steroids molecules (12). Steroidogenesis occurring in granulosa and cumulus cells affects oocyte development by improving the cytoplasmic and nuclear maturation (12). The higher expression of *StAR* mRNA in the FTML group indicates that co-culturing with ampullary cells might affect oocyte maturation and development by assistance in steroidogenesis. It has been shown that the addition of growth hormone to IVM medium increased mRNA expression levels of *StAR* in macaque MII oocytes, supporting the notion that growth hormone improves oocyte developmental competence by the steroidogenesis pathway (26). *StAR* protein also affects germ cell apoptosis. It has been shown that the suppression of the expression of *StAR* protein in Leydig cells increases apoptosis, resulting in impaired Leydig cell function in rats (27). Oocyte apoptosis might be a good marker for oocyte quality and development competency. Caspase-3 is the key caspase family member playing crucial roles in many cell apoptosis events. In addition, it is frequently used as the molecular marker to evaluate apoptosis (28). However, in the current study, *CASP3* gene expression in different groups was not different.

## 5. Conclusion

Using ampullary cells co-culture for IVM of bovine oocytes yields encouraging results and demonstrates the beneficial effect of this co-culture system on gene expression and developmental competence of bovine oocytes.

##  Conflict of Interest

The authors declare no conflict of interest.
